# Is strigolactone signaling a key player in regulating tiller formation in response to nitrogen?

**DOI:** 10.3389/fpls.2022.1081740

**Published:** 2022-12-15

**Authors:** Le Luo

**Affiliations:** MOA Key Laboratory of Plant Nutrition and Fertilization in Lower-Middle Reaches of the Yangtze River and State Key Laboratory of Crop Genetics and Germplasm Enhancement, Nanjing Agricultural University, Nanjing, China

**Keywords:** nitrogen, tiller, strigolactones, rice, signal, plant hormone

## Introduction

Rice (*Oryza sativa* L.) is one of the most important food crops, feeding half of the world’s population. Its yield relies on its agronomy traits, such as the number of panicles (also known as effective tiller), the number of grains per panicle, and the grain weight. Uncovering and elucidating the mechanism which could influence or regulate rice tiller developments is thus interesting, because it would provide a theoretical foundation for yield promotion.

Rice tillers are developed from the tiller buds located in the axils of leaves. The tiller bud is composed of the axillary meristem and the leaf primordia generating from the lateral meristem. Some of the tiller buds (including effective tillers and ineffective tillers) are activated to grow and form new leaf primordia and finally develop as tillers bearing panicles, whereas other tiller buds are found to stop growing after formation, without elongation, but in dormancy (Oikawa and Kyozuka, 2009). Recently, many genes regulating the growth and development of tiller buds in rice have been identified, so the regulatory mechanism and factors underlying tiller bud growth has gradually elucidated. Among these factors, plant hormone strigolactone (SL) and its biosynthesis and signaling pathway genes play the most prominent role in dominating rice tiller formation (Umehara et al., 2008; Gomez-Roldan et al., 2008). On the other hand, the growth of tiller bud is affected not only by the endogenous hormone signal but also by the environmental factors largely, such as water and nutrition.

Nitrogen (N) is one of the essential macronutrients needed by all plant species, and N deficiency hinders plant growth, thus decreasing yield (Stitt and Krapp, 1999; Good et al., 2004). A study on rice tiller formation has shown that the number of tillers in rice plant was decreased significantly under low-N conditions, whereas it could be significantly increased with sufficient N supply. To date, several genes coding such as nitrate transporters and transcription factors (TFs) have been identified as they are relevant in tiller formation in response to N supplies in the form of nitrate (NO_3_^−^) and ammonium (NH_4_^+^) mostly (Liu et al., 2021).

Although SL plays the key role in regulating tiller formation, little is known whether its biosynthesis and signaling genes are involved in N-dependent tiller formation. Therefore, in this opinion paper, we highlighted the emerging evidence to support a potential role of SL in mediating tiller formation in response to N availability. It further allows expanding the knowledge of the molecular mechanisms underlying tiller formation in response to environmental signals.

## Nitrogen regulation of tiller number

Application of N fertilizer can accelerate the growth and increase the number of rice tillers, when excessive N application decreases the number of effective tillers (Haque and Haque, 2016). Nitrate/peptide transporter family (NPF/NRT) genes have been reported to regulate tiller number and panicle structure *via* modulating nitrogen absorption and transport (Huang et al., 2018; Wang et al., 2018; Huang W.T. et al., 2019). Overexpression of *NRT2.3b*, which encodes a nitrate transporter, increased the panicle length, number of branches, number of seeds per panicle, and seed setting rate (Fan et al., 2016). *NPF7.7* can increase the inflow of both NO_3_^−^ and NH_4_^+^, thereby promoting the number of effective tillers and effective panicles of rice and grain yield (Huang et al., 2018). *NPF7.1* overexpression or *NPF7.4* knockout could promote rice axillary buds’ outgrowth, thereby increasing the number of tillers in rice (Huang W.T. et al., 2019). Additionally, the low-affinity nitrate transporter *NPF7.2* can activate the cell division of tiller buds, thereby increasing the number of tillers and grain yield (Wang et al., 2018).

Studies on amino acid transporters (AATs/AAPs) have also suggested that they are involved in the regulation of tiller development. Knocking out *AAP3* can promote the growth of buds and the number of effective tillers, thereby increasing rice grain yield; *AAP3* overexpression leads to the accumulation of amino acids *in vivo* but inhibits the growth of tiller buds (Lu et al., 2018). *OsAAP5* can affect rice tiller number and yield by regulating cytokinin (CK) biosynthesis (Wang et al., 2019). The amino acid biosynthesis genes are also involved in the regulation of tiller growth. The mutant of *asparagine synthetase 1* (*ASN1*) in rice reduces the concentration of asparagine and inhibits tiller bud outgrowth, hence restraining the number of tillers. It indicates that *ASN1* is involved in the bioprocess regulating rice tiller development (Luo et al., 2019b). Glutamine synthetase GS1;2 functions in the primary assimilation of NH_4_^+^ and promotes the growth of tiller buds by regulating N-dependent biosynthesis of phytohormone cytokinin (Ohashi et al., 2017).

In addition to cytokinin, other phytohormones and several TFs are also involved into the process of which N regulates tiller outgrowth deeply. For instance, the gibberellin (GA) signaling pathway was identified to be essential for N regulation on tiller number (Wu et al., 2020). GA can reduce the epigenetic modification and induce the expression of target genes by promoting the degradation of APETALA2-domain transcription factor NGR5, resulting in the inhibition of N-induced growth and development of tiller buds; the excellent allele *GRF4 ngr2* can largely increase tiller number and thus result in high NUE in the current high-yielding rice (Li et al., 2018). Meanwhile, a MADS box transcription factor OsMADS57, which is induced by nitrate, interacts with TEOSINTE BRANCHED1 (TB1) and Dwarf14 (D14) to control the growth of axillary buds (Guo et al., 2013; Huang S. J. et al., 2019). Most recently, through a genome-wide association study on nitrogen use efficiency and N regulation of tiller number, TCP19, encoding a TCP transcription factor family member, was identified to inhibit N-regulated tillering by promoting *DLT* expression (Liu et al., 2021). The nitrogen-induced LATERAL ORGAN BOUNDARIES DOMAIN (LBD) proteins OsLBD37 and OsLBD39 can directly bind to the promoter of *OsTCP19* and inhibit its activity, and *OsTCP19*, in turn, regulates tiller number and N use efficiency (NUE).

## Strigolactone plays a central role in the gene regulatory network of rice tiller development

In rice, SLs are biosynthesized from β-carotene, which is converted to carlactone (CL) by β-carotene isomerases CAROTINOID CLEAVAGE DEOXYGENASE 7 (CCD7) and CCD8, encoded by *DWARF17* (*D17*) and *D10*, respectively (Zou et al., 2006; Arite et al., 2007; Lin et al., 2009; Lopez-Obando et al., 2015; Waters et al., 2017). CL is further catalyzed to several types of SLs (Zhang et al., 2014). Finally, the SLs are percepted by DWARF14 (D14), which triggers the formation of a complex of D14, D3, and D53, leading to ubiquitination of D53 by the action of D3 and downstream genes (Arite et al., 2009; Gao et al., 2009; Liu et al., 2009; Jiang et al., 2013; Zhou et al., 2013; Lopez-Obando et al., 2015; Waters et al., 2017). Importantly, the mutants of SL biosynthesis genes *D10* and *D17* and SL signaling genes *D14*, *D3*, and *D53* all have increased the tiller number, suggesting a negative regulation role of SL on tiller number.

So far, several important factors dominating rice tiller development have been found to interact with the SL pathway. For instance, the well-known tiller-number suppressor, TB1, interacts with MADS57 to regulate tiller number by downregulating the expression of SL receptor gene *D14* (Guo et al., 2013). *Ideal Plant Architecture 1* (*IPA1*), which encodes a SQUAMOSA PROMOTER BINDING PROTEIN-LIKE (SPL) family TF *SPL14*, plays a significant role regulating rice plant architecture (Jiao et al., 2010; Miura et al., 2010). Later, it was identified as a downstream gene of the SL signal pathway and relevant to tiller number (Song et al., 2017). IPA1 can directly interact with D53, the key repressor of the SL signaling, and *IPA1* expression is suppressed by D53. On the other hand, IPA1 can bind to the promoter of *D53* and activate its expression, suggesting a feedback regulation between IPA1 and SL (Song et al., 2017).

Previous studies also suggest crosstalk between SL and other plant hormones during rice tiller development. For example, auxin has similar negative regulation on tiller number as SL, which suggests auxin may act in the upstream of the SL pathway. This is supported by evidence that auxin can induce the expressions of SL biosynthesis genes *MAX3*/*D17* and *MAX4*/*D10* (Zha et al., 2019). GA has been found to regulate tiller bud elongation *via* suppressing SL biosynthesis, which depends on GA receptor GID1 and F-box protein GID2 (Ito et al., 2017). Meanwhile, DELLA protein SLENDER RICE 1 (SLR1), one suppressor of GA signaling, promotes tiller number by interacting with D14 in an SL-dependent manner (Nakamura et al., 2013). Genetic evidence also suggests that SL and brassinosteroids (BRs) coordinately regulate rice tillering *via* activating the D53–BZR1 signal complex which bind the promoter of TB1 and repress its transcription (Fang et al., 2020). On the other hand, there is also crosstalk between SLs and abscisic acid (ABA) during rice tillering and plant adaptability to the environment (Ruyter-Spira et al., 2013; Luo et al.,2019a).

Under phosphorus (P) deficiency conditions, endogenous SL content is elevated in wild-type seedlings, leading to attenuated tiller bud outgrowth. However, this inhibition does not occur in the SL signaling mutant (*d3*) and biosynthesis mutant (*d10*). It indicates that SL signaling is involved in the coordination of tiller development and P metabolism (Umehara et al., 2010). A recent study also revealed that the SL signal pathway is involved in circadian-clock-regulated tiller bud and panicle development in rice (Wang et al., 2020). The *D14* gene encoding SL receptor is transcriptionally induced by CIRCADIAN CLOCK ASSOCIATED1 (CCA1), a core regulator of the circadian clock, to repress the tiller number, whereas PSEUDORESPONSE REGULATOR1 (PPR1), the suppressor of the circadian clock, causes the opposite effects on *D14* expression and tiller development.

## The potential role of strigolactones on nitrogen regulation of tiller development

The biosynthesis of SL is influenced largely by environmental factors, such as nutrients in soil. The increased level of SLs was also reported in response to P and N deficiency in many different species. Yoneyama et al. (2007a) found that the nature SL orobanchol exudate from red clover (*Trifolium pretense* L.) was elevated in response to various nutrients (P, N, K, Ca, and Mg) and, in sorghum, the major SL 5-deoxystrigol was much higher under low P and also N (Yoneyama et al., 2007b). A further study mainly focused on P which is involved in the SL regulation of tiller development. Under P-deficient conditions, the expression levels of SL biosynthesis genes were upregulated, resulting in the increase in 2′-epi-5-DS levels in rice seedlings. However, this effect was not observed in the SL mutants *d3* and *d10* (Umehara et al., 2010). Further studies suggested that the P levels are the real trigger of SL induction, whereas the N effect on SL levels depends on the type of plant, type of nutrient, degree of nutrient stress, and macronutrient uptake strategy (Yoneyama et al., 2012; Yoneyama et al., 2013).

Furthermore, the SL signal has been recently found to mediate the regulation of N in rice root development. N deficiency led to an increased SL content in rice root tissues, and N deficiency-induced root responses (i.e., increased seminal root length and reduced lateral root density) were significantly suppressed in SL biosynthetic and signaling mutants *d10*, *d27*, and *d3* (Umehara et al., 2010; Sun et al., 2014).

Because N is also an important restriction factor for the tiller outgrowth, its regulation on SL was also investigated. Under nitrogen-deficient conditions, the tiller number reduced in both WT and *d3* and *d10* mutants. However, the reduction was much severe in WT, which indicated that the responses to the N level was insensitive in the SL mutant compared with WT (Luo et al., 2018). To some extent, this phenomenon is like what occurred under P-deficient conditions. However, in N-deficient conditions, even at an early stage, the restriction of tiller outgrowth appeared. This is possible due to the different observation systems or the regulation pathways. SLs could also influence the translocation of N which was proven with the altered N metabolic genes between WT and *d* mutants (Luo et al., 2018). The restriction of tiller bud outgrowth under N-deficient conditions was possibly caused by the SL biosynthesis which was enhanced by the N deficiency; it could also be caused by the influenced N translocation which is regulated by SL as a signal.

## Prospects

**Figure 1:**
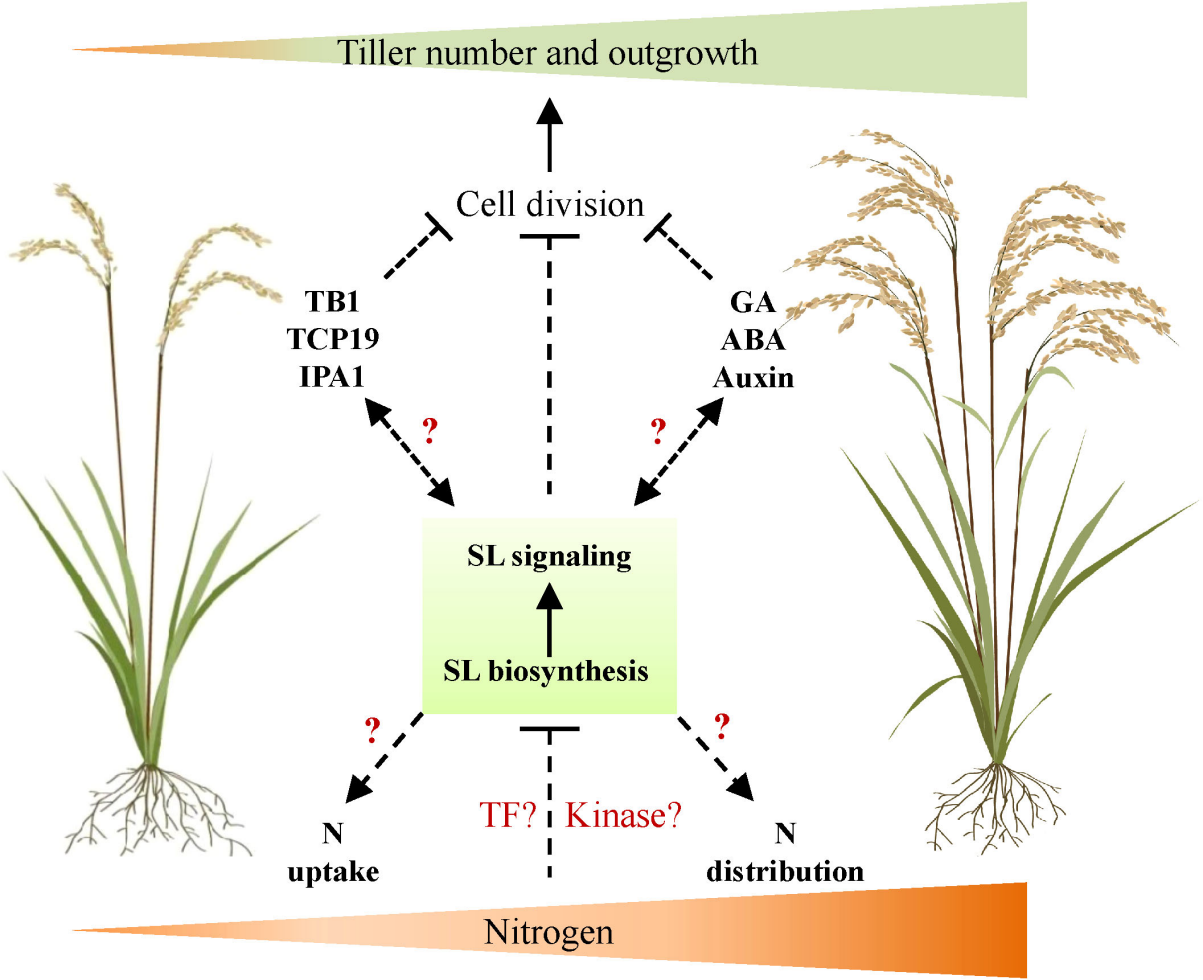
A proposed working model of SL signal on regulating rice tiller development in response to nitrogen. In this model, nitrogen supply increases rice tiller number and outgrowth through the negative regulation of SL biosynthesis and signaling transduction. However, it is uncharacterized whether specific transcription factors or kinases are activated by N to regulate SL signaling. Meanwhile, SL may also interact with well-known regulators of tiller number (e.g., TB1, TCP19, and IPA1) or plant hormones (e.g., GA, ABA, and auxin) to regulate the tiller number in response to external N supply. Furthermore, SL may also affect N uptake by root and N distribution in plant to regulate tiller number. TB1, TEOSINTE BRANCHED1; TCP19, TCP transcription factor 19; IPA1, Ideal Plant Architecture 1; N, nitrogen; TF, transcription factor; SL, strigolactone; GA, gibberellin; ABA, abscisic acid.

Tiller number is one of the important parameters and agronomy traits determining rice NUE and yield. Understanding the mechanism of its response to N availability is thus necessary and indispensable for high-efficiency agriculture in future. As the core regulator of tiller development, the SL signal is essential in mediating tiller development responses to various endogenous and environmental signals. This is based on promising evidence that several regulators, such as GA signal and TCP transcription factors, which mediate N-dependent tiller development, also interact with SL signaling components. Thus, we proposed a potential role of SL signals in regulating tiller development in response to the available N concentration underground, probably through the activation of the complex signaling cascades (**Figure 1**). Further investigation could be applied as, for instance, genetic approaches to explore SL signaling components that specifically regulate N-dependent tiller development and NUE; the possible molecular link between well-known tiller regulators and SL signals may be clarified.

## Author contributions

The author contributed to the writing of the manuscript, to designing the figure, and to its approval for publication.
